# Crosstalk between Metabolic Disorders and Immune Cells

**DOI:** 10.3390/ijms221810017

**Published:** 2021-09-16

**Authors:** Shinichi Saitoh, Koen Van Wijk, Osamu Nakajima

**Affiliations:** 1 Department of Immunology, Yamagata University Faculty of Medicine, Yamagata 990-9585, Japan; s-saitoh@med.id.yamagata-u.ac.jp; 2 Research Center for Molecular Genetics, Institute for Promotion of Medical Science Research, Yamagata University Faculty of Medicine, Yamagata 990-9585, Japan; koeninjapan@gmail.com

**Keywords:** chronic inflammation, obesity, M1/M2 macrophages, mesenchymal stem cells, CD4+ T cells, natural killer cells, innate lymphoid cells, cytokine, non-obese metabolic disorder, 5-aminolevulinic acid

## Abstract

Metabolic syndrome results from multiple risk factors that arise from insulin resistance induced by abnormal fat deposition. Chronic inflammation owing to obesity primarily results from the recruitment of pro-inflammatory M1 macrophages into the adipose tissue stroma, as the adipocytes within become hypertrophied. During obesity-induced inflammation in adipose tissue, pro-inflammatory cytokines are produced by macrophages and recruit further pro-inflammatory immune cells into the adipose tissue to boost the immune response. Here, we provide an overview of the biology of macrophages in adipose tissue and the relationship between other immune cells, such as CD4+ T cells, natural killer cells, and innate lymphoid cells, and obesity and type 2 diabetes. Finally, we discuss the link between the human pathology and immune response and metabolism and further highlight potential therapeutic targets for the treatment of metabolic disorders.

## 1. Chronic Inflammation and Obesity

Chronic inflammation owing to obesity primarily results from the recruitment of pro-inflammatory M1 macrophages into the adipose tissue stroma, as the adipocytes within become hypertrophied. Adipose tissue is responsible for energy metabolism regulation; it stores excess energy as neutral fat during overnutrition and supplies energy by lipolysis during nutritional deficiency. However, adipose tissue is not just a storage site, it is also an endocrine organ that secretes various adipose tissue-derived hormones called adipokines or adipocytokines. In times of ubiquitous nutritional plenty, as is frequent in the modern day, the energy storage function of adipose tissue contributes to obesity and is involved in various pathologies. Obesity negatively affects adipose tissue in many ways. It impairs its endocrine function, disrupts its regulatory function and adipokine production, and induces chronic inflammation. In addition, inflammatory adipokines secreted from adipose tissue can also act on distant organs, such as the liver and skeletal muscle, causing insulin resistance. The chronic inflammation caused by obesity is thought to be the underlying pathology of type 2 diabetes and its complications, such as retinal diseases, neuropathy, arteriosclerosis, dementia, and vulnerability to infection.

## 2. Biology of Macrophages in Adipose Tissue

### 2.1. Adipose Tissue-Resident Macrophages

Various immune cells are present in the adipose tissue of those suffering from obesity. Macrophages were the first immune cells to be discovered within adipose tissue [1]. Macrophages are classified into M1 and M2 macrophages based on their roles. Among other differences, M1 macrophages have a pro-inflammatory phenotype, whereas M2 macrophages have an anti-inflammatory phenotype. In non-obese adipose tissue, macrophages exhibiting M2-like markers, such as CD206, are the predominant subtype and highly express anti-inflammatory cytokines, such as IL-10 and TGF-β [2,3]. A study on fat apoptosis through targeted activation of a caspase-8 mouse model, which results in loss of adipocytes and reduction of adipose tissue weight, has reported a large influx of macrophages into the adipose tissue [4]. Similarly, studies in mice with β3-adrenergic receptor agonist treatment have shown that induction of adipogenesis triggers apoptosis in adipocytes, and that M2 macrophages are concentrated at sites undergoing apoptosis [5]. From these results, M2 macrophages are thought to be involved in the processing of tissue repair, the remodeling of adipose tissue, and the maintenance of insulin sensitivity.

Surprisingly, recent studies have reported contradictory results with respect to mediation of insulin resistance by M2 macrophages. Studies in CD206 DTR transgenic mice that express human diphtheria toxin (DT) receptors under the CD206 gene have demonstrated that DT administration specifically reduces the number of CD206+ M2-like macrophages. It also selectively reduces the expression of M2 markers, but not the expression of M1 markers [6,7]. Furthermore, M2 macrophage depletion improves both glucose tolerance and insulin sensitivity in lean and obese mice and induces the proliferation of adipocyte progenitors in a TGFβ-dependent manner [7,8]. Thus, in addition to their anti-inflammatory function in adipose tissue, M2 macrophages may have an adaptive mechanism for cellular restoration and contribute to the exacerbation of insulin resistance and type 2 diabetes.

On the other hand, M1 macrophages, which are derived from bone marrow and have increased expression in obesity, secrete inflammatory cytokines to induce insulin resistance. This recruitment of M1 macrophages into adipose tissue, and the resulting hypertrophy, are responsible for obesity-induced chronic inflammation [9]. M1 macrophage-derived tumor necrosis factor alpha (TNF-α) induces inflammatory cytokine production in adipocytes and promotes lipolysis in hypertrophied adipocytes [10]. As a result, free fatty acids (FFAs), especially saturated fatty acids (SFAs), are released locally in adipose tissue and have been reported to activate the toll-like receptor TLR4 on macrophages, which leads to the activation of the NF-κB pathway and further expression of pro-inflammatory cytokines. However, recent studies have argued against conventional mechanisms by which SFAs can bind to and activate TLR4 [11]. Instead, long-chain SFA palmitate is implicated as a direct TLR4 agonist. TLR4 does, however, indirectly regulate SFA-induced inflammation by altering macrophage lipid metabolism [12,13,14].

As mentioned above, it is well known that macrophage populations display phenotypic heterogeneity and high plasticity according to extracellular environmental conditions and intracellular signaling [15,16,17]. When enhancing aerobic glycolysis mediated by mTOR/HIF-1 signaling, M1 macrophages are polarized and activated by pro-inflammatory stimuli such as lipopolysaccharide (LPS) and IFN-γ, and exhibit increased aerobic glycolysis through mTOR/HIF-1 signaling [18,19]. In contrast, M2 macrophages are often polarized and enhanced by T-helper 2 (Th2) cytokines such as IL-4 and IL-13 and exhibit an increased mitochondrial metabolism through oxidative phosphorylation (OXPHOS)- and STAT6/PPARγ-mediated fatty acid oxidation [20,21,22]. Thus, mitochondrial metabolism regulates immune cell function. In particular, the transition between aerobic glycolysis and OXPHOS plays a fundamental role in macrophage polarization.

### 2.2. Adipose Tissue-Derived Mesenchymal Stem Cells

**Figure 1:**
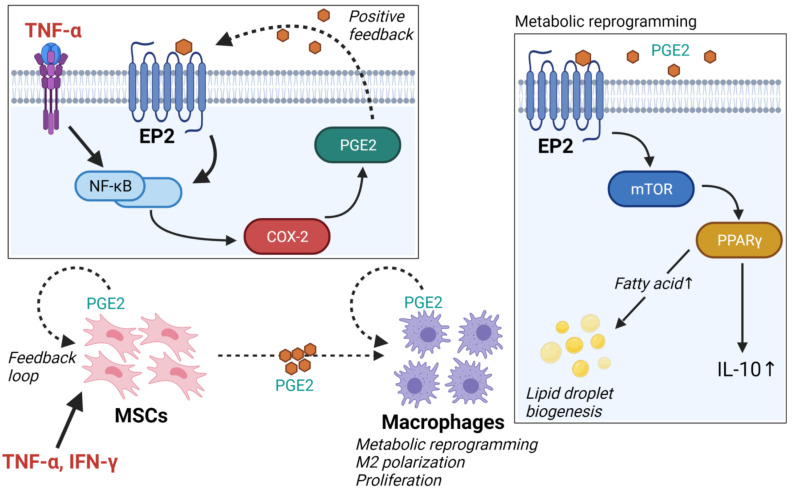
Schematic representation of the crosstalk between mesenchymal stem cells (MSCs) and macrophages. Pro-inflammatory cytokines such as TNF-α secreted by macrophages stimulate MSCs to produce and secrete prostaglandin E2 (PGE2). MSC-secreted PGE2 promotes lipid droplet biogenesis in macrophages and mediates macrophage polarization. PGE-2-mediated activation of the EP2 receptor induces additional COX-2 expression, further amplifying downstream PGE2 generation. The figure was created using BioRender.com (accessed on 14 September 2021).

Recently, the crosstalk between mesenchymal stem cells (MSCs) and macrophages has attracted attention because of its potential therapeutic benefits [23]. Interestingly, human MSCs and primary macrophage co-culture experiments have demonstrated that MSC-secreted PGE2 mediates macrophage polarization by modulating metabolism [24,25]. Furthermore, adipose tissue-derived MSCs also promote lipid droplet biogenesis in macrophages and activate the mTOR/PPARγ pathway [26,27], which leads to COX-2 expression and, consequently, PGE2 production [28,29]. The binding of PGE2 to the G-protein-coupled receptors EP1–EP4, leads to the activation of different downstream signaling pathways: among them, PGE2/EP2 signaling. This pathway, via transcription of NF-κB, has a pro-inflammatory effect [30,31,32]. In aged mice, PGE2/EP2 signaling has a specific suppressive effect on both aerobic glycolysis and OXPHOS in glucose metabolism, leading to immunometabolic dysfunction and chronic inflammation [33]. These findings are consistent with the positive feedback PGE2/EP2 signaling hypothesis, in which the PGE2-mediated activation of the EP2 receptor induces additional COX-2 expression, further amplifying downstream PGE2 generation (Figure 1) [31,34]. Inhibition of PGE2/EP2 signaling has, therefore, become a new drug target for the development of anti-aging therapies.

It is known that adipose tissue-derived MSCs produce angiogenic molecules [35]. The vascular endothelial growth factor (VEGF) plays a role in the formation of blood vessels and the remodeling of the vasculature. Systemic lupus erythematosus (SLE) patients have the highest serum VEGF levels [36], and the serum VEGF level was significantly increased in diabetic patients, especially in those with vascular complications [37]. Moreover, recent findings suggest that exsomes from human adipose tissue-derived MSCs possess a higher capacity to enhance angiogenesis via VEGF/VEGF-R signaling [38].

## 3. Adipose Tissue-Resident Lymphocytes

During obesity-induced inflammation in adipose tissue, pro-inflammatory cytokines such as TNF-α are produced by macrophages and recruit further pro-inflammatory immune cells, such as CD4+ T cells, natural killer (NK) cells, and innate lymphoid cells, into the adipose tissue to boost the immune response.

### 3.1. CD4+ T Cells

The vast majority of studies on CD4+ T cells has been conducted on cells in the primary and secondary lymphoid tissues, and the biology of these cells has been well understood. However, recent studies have revealed unique features of the biology of adipose tissue-resident CD4+ cells [39]. Studies in mouse adipose tissue showed that the CD4+ CD25+ Foxp3+ T regulatory cell (Treg) population resides in normal lean adipose tissue and mediates the induction of alternatively activated macrophages to maintain an anti-inflammatory state. Surprisingly, the proportion of Tregs expressing Foxp3 reached high frequencies of up to 30–40% of the total CD4+ T cell population in lean adipose tissue, and this accumulation continued with increasing age until maturity, peaking at an age of above 20–25 weeks [40,41]. Loss of function experiments using mice expressing the diphtheria toxin receptor (DTR) under the Foxp3 regulatory elements revealed that the depletion of Tregs results in impaired metabolic parameters such as increased fasting blood glucose levels and insulin resistance [42]. This result identifies an important role of these cells and provides deeper insight into the reason behind the strong accumulation of Tregs in adipose tissue. The unique features and roles of Tregs have significance for further study of metabolic health.

To examine the physiological function of adipose tissue-resident immune cells in humans, many studies have begun to compare biopsy samples from lean and obese subjects. Focusing on T cells, the proportion of CD4+ or CD8+ T cells in subcutaneous adipose tissue samples ranges from 0.5 to 5% of the stromal vascular fraction (SVF), but no correlation has been found between the population and the degree of obesity [43,44]. Rather, when assessing the level of T lymphocyte activation, positive correlations were found instead between waist circumference and the expression levels of CD69 and CD25 on activated adipose tissue-resident CD4+ and CD8+ T cells [43]. In line with these findings, Foxp3 mRNA expression levels in adipose tissue-resident CD4+ T cells were found to be elevated and correlated with the degree of obesity. In addition, CD4+ helper T cells isolated from human peripheral blood mononuclear cells (PBMCs) cultured in conditioned medium from human adipose tissue-derived MSCs, were found to have an increased expression of Foxp3 and IL-10 [45]. Recent studies have also reported that co-culture with human adipose-derived MSCs increases the proportion of CD4+ CD25+ Foxp3+ Tregs in PBMCs in a dose-dependent manner [46]. These findings suggest that, during obesity, adipose tissue-resident immune cells could limit pro-inflammatory processes within adipocytes and induce anti-inflammatory effects in adipose tissue.

### 3.2. Natural Killer Cells

**Figure 2:**
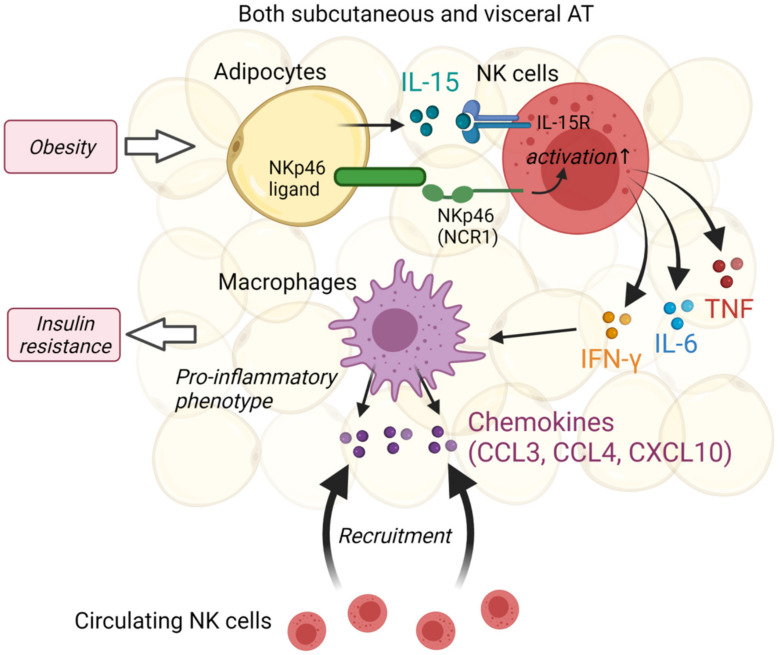
Schematic representation of the function of NK cells in adipose tissue homeostasis. Both SAT and VAT express IL-15 and IL-15Rα, and IL-15 levels are higher in obese patients than in healthy patients. Obesity induces the upregulation of NKp46 (NCR1) ligand expression in adipocytes, which activates adipose tissue-resident NK cells to rapidly increase IFN-γ production, thereby promoting the accumulation of pro-inflammatory M1 macrophages and insulin resistance. Macrophages increase the production of chemokines and let them further enhance NK cells recruitment. The figure was created using BioRender.com (accessed on 14 September 2021).

NK cells are a subset of innate lymphoid cells that respond to virus-infected cells and play an important role in anti-tumor immunity. Several recent studies have highlighted the important role of NK cells in adipose tissue homeostasis and insulin resistance; however, the initial experimental models and assumptions of the relationship between NK cells and adipose tissue in those studies have been previously described. O’Rourke reported that NK cells in human subcutaneous adipose tissue (SAT) showed more activation features than NK cells from the blood, and consequently, the first evidence that NK cells could regulate adipose tissue macrophages [47]. Recent studies have suggested that NK cells play a potential role in the inflammatory cascade in adipose tissue. This cascade is mediated by adipocyte-derived IL-15 and includes inflammatory cytokine secretion in NK cells, promoting macrophage polarization to a pro-inflammatory phenotype, and consequently, the inducing insulin resistance [48] (Figure 2). NK cell development, homeostasis, and function require IL-15 and its complex with IL-15 receptor alpha (IL-15Rα). Investigations of IL-15 and IL-15Rα in human adipose tissue found that both SAT and visceral adipose tissue (VAT) produced IL-15 and IL-15Rα RNA, and SAT IL-15 levels were higher in obese patients than in healthy patients and correlated with SAT lipolysis. The VAT stromal vascular fraction (SVF), which could be more associated with inflammation by macrophages than SAT, expressed more RNA than adipocytes [49,50]. These findings suggest that IL-15 and IL-15Rα derived adipose tissue-resident immune cells may support NK cell function via paracrine and autocrine mechanisms.

NCR1 (natural cytotoxicity triggering receptor), known as NKp46 in humans, is an activating receptor expressed almost entirely on NK cells that functions as a detector of virally infected and cancerous cells. Moreover, NCR1 and other NK cell activating receptors (such as NKG2D) could contribute to protection against auto-immunity or to tumor immune escape [51]. In line with this, a reduction of these inhibitory NK receptors is observed in HIV-infected patients treated by highly active anti-retroviral therapy (HAART) [52]. In addition to these well-established functions, a study using high-fat diet (HFD)-fed and NCR1 deficient mice demonstrated that obesity induced the upregulation of NCR1 ligand expression in the VAT, which activates adipose tissue-resident NK cells to rapidly increase IFN-γ production, thereby promoting the accumulation of pro-inflammatory M1 macrophages and insulin resistance [53] (Figure 2). 

In addition, NK cell suppression using either neutralizing antibodies or E4bp4 heterozygous knockout mice was found to improve adipose tissue macrophage inflammation and obesity-induced insulin resistance as well as to decrease the expression of pro-inflammatory cytokines such as IL-6 and TNF, which are elevated in the VAT of HFD-fed mice [54]. With elevated pro-inflammatory cytokines, HFD also induced the macrophage chemokine Ccl2 expression in VAT and sorted adipose tissue macrophages from HFD mice increased the production of CCL3, CCL4, and CXCL10, known chemoattractants for NK cells, and let them further enhance their recruitment. These findings show that adipose tissue-resident NK cells produce IL-6, TNF, and IFN-γ, which are characteristic cytokines of NK cells, and regulate adipose tissue inflammation by macrophage recruitment. Conversely, inflamed macrophages secrete chemoattractants for NK cells and promote NK cell activation and recruitment [55,56]. This suggestion is consistent with the human findings of IL-15 and IL-15Rα [51,52].

Adipocytes are the main constituent cells of adipose tissue and play an important role in innate responses through the secretion of a variety of a adipokines such as adiponectin and leptin [57,58]. Adiponectin and leptin have dual roles as hormones and cytokines; as hormones, they regulate glucose levels, lipid metabolism, and energy homeostasis; as cytokines, they regulate inflammatory responses [59]. Investigation on the serum adiponectin levels in patients with T2DM who were overweight or non-overweight showed that patients with T2DM had lower levels of adiponectin and IL-10 and higher levels of TNF-α and IL-1β. Moreover, the adiponectin levels were correlated with cytokine levels and the clinical parameters of overweight and T2DM [60]. Leptin plays a prominent role in innate responses, such as the activation of granulocytes and macrophages, M1/M2 polarization, and especially, the activation of NK cell cytotoxicity [61]. Leptin treatment for isolated human NK cells increases cytotoxicity against the K562 erythroleukemia cell line and IFN-γ production [62]. Furthermore, investigation of the effect of exercise training and nutritional intervention for healthy adults with obesity demonstrated that IFN-γ expression in NK cells increased after weight loss and plasma leptin decreased after intervention [63].

### 3.3. Innate Lymphoid Cells

Innate lymphoid cells (ILC) are tissue-resident cells that contribute to tissue repair and react early to defend against invading pathogens. Conventionally, NK cells are categorized as group 1 innate lymphoid cells (ILC1) with phenotypic and functional similarities [64]. However, a recent study has suggested that adipose tissue-resident ILC1s are phenotypically and functionally distinct from adipose NK cells, where adipose tissue-resident ILC1 proliferates and accumulates within the SAT of HFD-fed mice in an IL-12R/STAT4-dependent manner and drives obesity-associated insulin resistance through pro-inflammatory M1 macrophage polarization [65].

**Figure 3:**
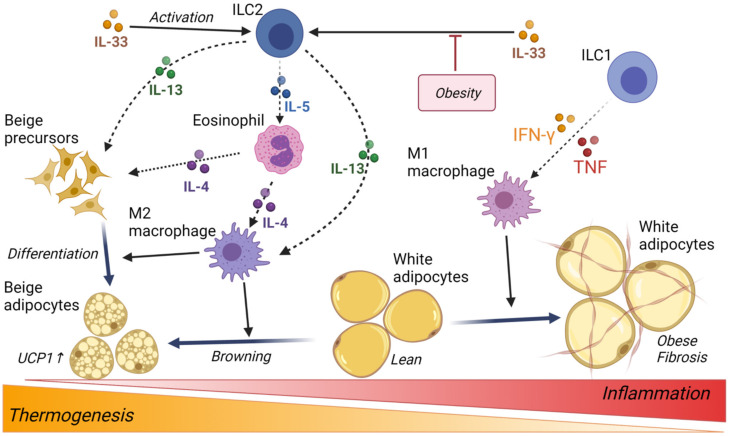
Schematic representation of the link between innate lymphoid cells and the regulation of adipocyte thermogenesis and obesity in WAT. Tissue-resident ILC1 promotes macrophage activation and adipose fibrosis. ILC2 respond to epithelial cell-derived cytokine IL-33. Activated ILC2 enhances proliferation and commitment of beige adipocyte precursors, where IL-13 and eosinophil-derived IL-4 promote the growth of beige fat via IL-4 receptor signaling. Eosinophils are a major source of IL-4, and they migrate into adipose tissue and play a role in the maintenance of adipose M2 macrophages. The figure was created using BioRender.com (accessed on 14 September 2021).

Notably, a recent human study was performed to test whether ILC contributes to the development of obese T2D, where the number of circulating and adipose tissue ILC1 was compared among obese patients who underwent gastric bypass surgery and non-obese non-T2D controls who underwent elective abdominal surgery [66]. Herein, adipose ILC1, which was identified as Lin− CD45+ CD127+ CD117− CRTH2− NKP44− lymphocytes, correlated with the development of obesity and obese T2D and also correlated with trichrome C staining areas indicating adipose tissue fibrosis. Furthermore, SVF from controls were co-cultured in vitro with ILC1 or ILC-depleted CD45+ cells sorted from the SVF of obese T2D patients, and the mRNA expression of Tgfb1, Mincle, inducible nitric oxide synthase (iNOS), and collagen genes, Col1a and Col3a, was increased in the SVF of the ILC non-depleted group. These findings suggest that human adipose tissue-resident ILC1 promotes macrophage activation and adipose fibrosis and may, therefore, be a novel therapeutic target for the treatment of obesity-induced type 2 diabetes (Figure 3).

Group 2 innate lymphoid cells (ILC2) respond to epithelial cell-derived cytokine IL-33, activate and accumulate in various organs and tissues, and then secrete IL-5 and IL-13 to initiate type 2 innate and adaptive immune responses. ILC2 has been identified in the white adipose tissue (WAT) of mice, which plays a role in the maintenance of M2-like macrophage and eosinophils to regulate metabolic homeostasis [67]. In human subcutaneous WAT, ILC2 was identified as a lineage negative, CD25 (IL-2Rα)-positive and CD127 (IL-7Rα)-positive cell populations, which expressed GATA-3 and IL-33R consistent with ILC2 in other tissues, and their frequencies in obese subjects were decreased compared with non-obese controls as well as in subcutaneous WAT of mice [68]. IL-33 plays a critical role in the maintenance and activation of ILC2 in WAT [68,69]. Activated ILC2 enhances the proliferation and commitment of beige adipocyte precursors, where IL-13 and eosinophil-derived IL-4 promote the growth of beige fat via IL-4 receptor signaling. Finally, the browning or beiging of WAT and white adipocytes stimulates thermogenesis and increases energy expenditure; thus, ILC2 in WAT has been implicated in the regulation of obesity and metabolic dysfunction [70] (Figure 3).

## 4. Adipocyte Thermogenesis and Browning

In contrast to white adipocytes, which store lipid depots of excess energy in the major form of triglycerides, brown and beige adipocytes are now recognized to play a potential role in anti-obesity function, which contributes to energy expenditure through diet- and cold-induced thermogenesis [71,72]. Several studies have highlighted the importance of anti-inflammatory cytokines, such as IL-4 and the closely related cytokine, IL-13, in regulating adipocyte thermogenesis [73,74]. For instance, genetic disruption of IL-4/13 signaling or eosinophils impairs cold-inducible beige adipocytes, and conversely, administration of IL-4 increases beige adipocytes to recover obesity in thermoneutral mice; that is, eosinophils and type 2 cytokine signaling in macrophages require thermogenic activation [73]. Furthermore, loss of IL-10 signaling increases energy expenditure and thermogenesis in adipocytes, which induces the formation of brown-like features in white adipocytes [75]. This is in contradiction to the well-known biology of IL-10 in adipose tissue, where M2 macrophages are predominant in non-obese adipose tissue and highly express anti-inflammatory cytokines [2,3]. Given that IL-10 plays an important role in the attenuation of inflammation, this result suggests that IL-10 signaling in adipocytes might contribute to promote lipid storage and maintain energy demand in the case of acute responses such as infection.

Thermogenesis is the main function of brown adipose tissue (BAT) and cellular dissipation of energy via the production of heat [76]. Using rodent models, it was demonstrated that deficient BAT thermogenesis is committed to the development of obesity, and activation of BAT thermogenesis reduces weight gain [77,78,79]. It is generally accepted that BAT thermogenesis is primarily mediated by the action of mitochondrial uncoupling protein 1 (UCP1) to protect against obesity and diabetes [80,81], which is a major focus in human obesity research [82,83,84]. Furthermore, eosinophils are a major source of IL-4, and they migrate into adipose tissue and play a role in the maintenance of adipose M2 macrophages associated with the correction of glucose metabolism [85] (Figure 3).

Fas, also known as CD95 or APO-1, is a death receptor that belongs to the TNF receptor family member. Fas expression was increased in adipocytes isolated from insulin-resistant mice and in the adipose tissues of obese and diabetic patients [86]. Fas mutant mice show a lean phenotype and prevent HFD-induced obesity by upregulating the expression levels of UCP1, IL-4, and IL-10 in WAT [87]. Thus, FAS/FAS ligand-mediated signaling appears to be more involved in controlling local inflammation in adipose tissue and promoting WAT browning, that is, TNF is associated with obesity via deactivation of thermogenesis [88,89].

## 5. Liver-Resident Natural Killer Cells

NK cell populations represent a small fraction of circulating lymphoid cells, but a large fraction of lymphoid cells in the liver (10–20% in mice and 40–50% in humans). NK cells also play an important role in liver disease and control. Non-alcoholic fatty liver disease (NAFLD) is the most common chronic liver disorder and is closely linked to obesity, in which excess depot fat accumulates in the liver. NAFLD ranges from simple steatosis to low-grade inflammatory stage known as non-alcoholic steatohepatitis (NASH), which has been observed in 20–30% of NAFLD cases. Diet-induced obesity leads to a reduction in NK cell cytotoxic function, which is followed by lipid accumulation in NK cells [90,91]. Metabolic reprogramming, especially glycolysis downregulation, impairs anti-tumor immunity in the liver, which is a potentially important role of NK cells against liver disease [92]. Consequently, in patients with advanced fibrosis and later stages of NASH, reduced cytotoxic activity in NK cells contributes to tumor cell growth. Recent studies have shown that NK cells in the liver of human NAFLD patients reduced degranulation as a marker of cytotoxicity [93]. In obese mice, hepatic NK cells showed a reduction in CD107a degranulation and cytotoxic immune function. In human NAFLD patients, NK cells isolated from the liver displayed a negative correlation with disease severity.

IL-15 is a key cytokine essential for innate immune cell homeostasis during obesity-induced liver inflammation and NASH development [94]. Most recently, an observational study in middle-aged adults showed that the serum IL-15 levels of obese patients with NAFLD were higher than those of healthy subjects and associated with the levels of monocyte chemoattractant protein-1 (Mcp-1) and the granulocyte-macrophage colony-stimulating factor (GM-CSF), which are well-known inflammatory biomarkers. Moreover, IL-15 levels appeared to be predictive factors for the disease progression in NAFLD patients [95]. The cellular source of IL-15 in the liver is hepatocytes. Hepatocytes constitute 60–80% of the total cell population in the liver and play many important roles in metabolism, detoxification, and protein synthesis [96]. Orthotopic liver transplantation experiments using IRF-1 knockout mice revealed that hepatocytes are major producers of soluble IL-15 and IL-15Rα complexes among liver cell populations [97]. A study using IL-15 knockout mice and IL-15Rα conditional knockout mice showed that hepatocyte-mediated IL-15 signaling by IL-15Rα expression is required to maintain the homeostasis of NK and NKT cells in the liver [98]. In turn, activated NK and NKT cells produce inflammatory cytokines such as IL-6, TNF, and IFN-γ. In particular, TNF-α was an important determinant for the pathogenesis of NAFLD, but on the other hand, IL-10 decreased by the progression of NAFLD [99]. The balance between pro- and anti-inflammatory cytokines is an important function and risk factor in the development of liver disease and NAFLD.

## 6. Links with Human Pathology

### 6.1. Metabolically Healthy Obesity

Approximately 30–40% of people with obesity are metabolically healthy obese individuals (MHO), when healthy is defined as the absence of metabolic disorders [100]. Research in MHO has provided insights into the complexity of the crosstalk between metabolic syndrome and chronic inflammation [101]. Studies in adipose tissue from MHO have demonstrated that the expression and secretion levels of TGF-β were increased, and in addition, the circulating level of IL-10 was higher in serum [102]. TGF-β and IL-10 are major anti-inflammatory cytokines and were previously shown to be required to suppress inflammation in adipose tissue. Furthermore, the expression of FOXP3, the central regulator of Treg cell development and function, was also greater in adipose tissue from MHO, which is consistent with other studies [102]. FOXP3+ Treg cells secrete IL-13 to stimulate IL-10 production in macrophages, which might be mediated by TGF-β [103,104]. Thus, MHO may be associated with an increased expression of FOXP3, IL-10, and TGF-β [105]. A recent study illustrated the possibility of activating the TGF-β/BMP signaling pathway in obese individuals without type 2 diabetes, as well as MHO, through the transcription analysis comparing metabolically unhealthy individuals by RNA-seq [106]. Here, RNA-seq of mRNA and microRNA was performed in adipose tissue from obese patients; therefore, SMAD4 and RUNX2 genes, which are components of TGFβ signals, and miRNAs associated with these genes increased their expression in obese individuals without type 2 diabetes. In turn, RUNX2 is a master regulator of osteogenic differentiation of MSCs, and SMAD4 is a pivotal regulator of the TGF-β pathway and is known as a tumor suppressor gene [107]. Elucidation of the regulator of adipocyte differentiation of MSCs is important for developing therapeutic strategies for both obesity and type 2 diabetes. Recent genetic approaches, such as this transcription analysis, will help in diagnostic purposes.

### 6.2. Non-Obese Metabolic Disorder

It is widely known that Asians are at an increased risk of developing diabetes even with mild obesity. A U.S. study reported that Asian Americans have a higher risk to develop T2DM than Caucasians with the same body mass index (BMI) [108]. In addition, a survey of non-obese individuals in Japan has revealed that insulin resistance in skeletal muscle increases when the risk of heart disease, such as hypertension and dyslipidemia, is also present [109], and non-obese Japanese men with a fatty liver alone had a lower insulin sensitivity than those with visceral fat accumulation alone, suggesting that insulin resistance may develop without fat accumulation in adipose tissue [110]. Elucidation of the pathophysiology of non-obese insulin resistance and type 2 diabetes is extremely important in the prevention of intervention in metabolic syndrome. However, in high-fat diet load and obesity models in laboratory animals, ectopic fat accumulation induces systemic lipotoxicity; consequently, systemic lipotoxicity causes further insulin dysfunction, making it difficult to unravel the inherent abnormalities. Thus, studies using experimental models or subjects with non-obese insulin resistance and type 2 diabetes may be the key to preventing or delaying diabetes-related complications.

5-Aminolevulinic acid (5-ALA) is a natural amino acid and the first precursor of heme biosynthesis. Notably, two recent cohort studies suggest that oral 5-ALA supplementation can protect against mild hyperglycemia and help prevent type-2 diabetes [111,112]; 5-ALA or heme is associated with glucose metabolism in vivo. To clarify the relationship between heme and glucose metabolism in vivo, we previously examined glucose metabolism in 5-ALA deficient mice, which had been obtained with 5-ALA synthase ALAS1 heterozygous (*Alas1*^+/−^) mice. Here, we showed that *Alas1*^+/−^ mice displayed insulin resistance, which was cured by 5-ALA administration. Despite the onset of insulin resistance, *Alas1*^+/−^ mice were not obese [113,114]. These results indicate that *Alas1*^+/−^ mice can be considered a non-obese diabetic model. Our earlier study, which reported the generation of *Alas1*^+/−^ mice, revealed that ALAS1 is moderately expressed in monocytes and scarcely expressed in lymphocytes among peripheral blood leukocytes; in particular, Alas1 is highly expressed in granulocytes, and this high expression has been observed until they fully differentiated into mature neutrophils [115]. To test whether Alas1 and 5-ALA have any potential effect on neutrophil function, we measured reactive oxygen species (ROS) production and phagocytosis in neutrophils from *Alas1*^+/−^ mice and mice after 5-ALA administration. *Alas1*^+/−^ mice exhibited decreased levels of ROS generated through the activation of NADPH oxidase, and 5-ALA administration increased ROS production in neutrophils; similar results were observed in phagocytosis [Saitoh, unpublished]. In line with this, in vitro analysis using the mouse macrophage cell line RAW264.7 showed that the addition of 5-ALA reduced the expression of pro-inflammatory cytokines with HO-1 induction [116], since the exogenous addition of 5-ALA induces HO-1 expression [117]. These findings demonstrate that *Alas1*^+/−^ mice are a novel model for non-obese diabetes, and 5-ALA is a potential therapeutic target against inflammation-induced insulin resistance.

### 6.3. Cytokine for Type 2 Diabetes Treatments

Interleukin-6 (IL-6) family is a group of cytokines consisting of IL-6, IL-11, oncostatin M, ciliary neurotrophic factor (CNTF), and leukemia inhibitory factor (LIF) and is defined by the requirement of glycoprotein 130 (gp130) as a signal-transducing subunit in the receptor complex cytokines [118]. Many studies have helped elucidate IL-6 function in adipose tissue, and it is now well known that IL-6 is one of the most important cytokines and a marker for inflammation, secreted from both adipose tissue-resident macrophages and adipocytes. However, the effect of IL-6 on metabolic disorders has been debated for several decades. Nonetheless, it is possible that IL-6 and IL-6 receptor (IL-6R) signaling in adipose tissue-resident macrophages may induce clinical inflammation and mediate insulin resistance.

Axokine, the recombinant human variant of CNTF, initially entered human clinical trials for the treatment of amyotrophic lateral sclerosis (ALS) but was repurposed to treat obesity and type 2 diabetes when treated patients were observed to lose body weight. After its initial clinical trials, in which treatment resulted in more weight loss than the placebo, some treated patients developed auto-antibodies, raising safety concerns [119], and the clinical development of axokines was discontinued. Although IL-6 protects against obesity and insulin resistance, it can also be pro-inflammatory owing to its trans signaling, in which IL-6 binds soluble IL-6R and then forms a heterotrimer with gp130 expressed on the cell surface. Since gp130 has an almost ubiquitous expression, almost all cells are responsive to trans signaling. Thus, the selectively blocking of IL-6 trans signaling is needed to prevent the pro-inflammatory effects of IL-6 signaling and develop drugs for inflammation-induced insulin resistance [120].

Treatment with soluble gp130Fc, in which Fc fusion is an established method used to prolong the half-life by fusing the fragment crystallizable region (Fc) of IgG to recombinant or therapeutic proteins, has demonstrated the blocking of IL-6 trans signaling, unlike complete ablation of IL-6 signaling, and efficacy against HFD-induced macrophage accumulation but no improvement of insulin resistance [121]. Most recently, the chimeric gp130 receptor ligand IC7Fc, which is structurally similar to IL-6 and contains three different binding sites against IL-6R, gp130, and the LIF receptor from CNTF, was designed and generated [122]. As treatment with IC7 did not involve signs of inflammation, IC7Fc is more likely to be an effective drug against type 2 diabetes. Here, IC7Fc injection in diet-induced obese mice reduced fat mass and fasting glucose and improved glucose tolerance, while maintaining lean mass through activation of YAP1 in skeletal muscles. Importantly, experiments in non-human primates, crab-eating macaques, mimicking a phase I human clinical trial, demonstrated that IC7Fc is safe with no adverse outcomes and a suite of markers of inflammation. These findings show that inflammatory cytokines, especially the IL-6 family, may be novel biological therapeutic targets for the treatment of type 2 diabetes and metabolic disorders, even when its safety for inflammation is guaranteed.

## 7. Conclusions

Metabolic syndrome involves a group of multiple risk factors that arise from insulin resistance induced by abnormal fat deposition. It comprises a combination of risk factors for cardiovascular disease and stroke, as well as diabetes, fatty liver, and several cancers. According to the World Health Organization, approximately 2 billion adults are overweight, of which 650 million are considered to be affected by obesity with a BMI over 30, which equates to 39% of adults aged 18 or above who are overweight, and 13% are obese. Currently, treatment of obesity is recognized as one of the most important public health tasks worldwide.

Since the crosstalk between metabolic disorders and immune cells originates from initial evidence for adipose tissue-resident macrophages, it is well known that obesity induces the accumulation of pro-inflammatory macrophages and chronic inflammation; in contrast, health and a normal metabolic state provide the physiological and adaptive plasticity of M1/M2 polarization. Over the past two decades, because almost all types of immune cells have become discriminable and sortable, the biology of tissue-resident lymphocytes has been revealed. Surprisingly, the central players in adaptive and innate immune responses, such as CD4+ T and NK cells, are strongly associated with obesity-induced inflammation and thermogenesis. Thermogenesis is a hallmark feature of adipose tissue and is fundamental to whole-body energy metabolism. Many recent studies of adipocyte progenitor cells have demonstrated that differentiation into brown and beige adipocyte and WAT browning have been regulated by anti-inflammatory cytokines from tissue-resident immune cells, and so could be potential therapeutic targets for the treatment of metabolic disorders [123,124].

## Data Availability

Not applicable.

## References

[1] Weisberg S.P., McCann D., Desai M., Rosenbaum M., Leibel R.L., Ferrante A.W. (2003). Obesity is associated with macrophage accumulation in adipose tissue. J. Clin. Investig..

[2] Hong E.G., Ko H.J., Cho Y.R., Kim H.J., Ma Z.X., Yu T.Y., Friedline R.H., Kurt-Jones E., Finberg R., Fischer M.A. (2009). Interleukin-10 Prevents Diet-Induced Insulin Resistance by Attenuating Macrophage and Cytokine Response in Skeletal Muscle. Diabetes.

[3] Gao M.M., Zhang C.B., Ma Y.J., Bu L., Yan L.N., Liu D.X. (2013). Hydrodynamic Delivery of mIL10 Gene Protects Mice from High-fat Diet-induced Obesity and Glucose Intolerance. Mol. Ther..

[4] Fischer-Posovszky P., Wang Q.A., Asterholm I.W., Rutkowski J.M., Scherer P.E. (2011). Targeted Deletion of Adipocytes by Apoptosis Leads to Adipose Tissue Recruitment of Alternatively Activated M2 Macrophages. Endocrinology.

[5] Lee Y.H., Petkova A.P., Granneman J.G. (2013). Identification of an Adipogenic Niche for Adipose Tissue Remodeling and Restoration. Cell Metab..

[6] Kambara K., Ohashi W., Tomita K., Takashina M., Fujisaka S., Hayashit R., Mori H., Tobe K., Hattori Y. (2015). In Vivo Depletion of CD206(+) M2 Macrophages Exaggerates Lung Injury in Endotoxemic Mice. Am. J. Pathol..

[7] Nawaz A., Aminuddin A., Kado T., Takikawa A., Yamamoto S., Tsuneyama K., Igarashi Y., Ikutani M., Nishida Y., Nagai Y. (2017). CD206(+) M2-like macrophages regulate systemic glucose metabolism by inhibiting proliferation of adipocyte progenitors. Nat. Commun..

[8] Soucie E.L., Weng Z.M., Geirsdottir L., Molawi K., Maurizio J., Fenouil R., Mossadegh-Keller N., Gimenez G., VanHille L., Beniazza M. (2016). Lineage-specific enhancers activate self-renewal genes in macrophages and embryonic stem cells. Science.

[9] Lumeng C.N., Bodzin J.L., Saltiel A.R. (2007). Obesity induces a phenotypic switch in adipose tissue macrophage polarization. J. Clin. Investig..

[10] Langin D., Arner P. (2006). Importance of TNF alpha and neutral lipases in human adipose tissue lipolysis. Trends Endocrinol. Metab..

[11] Lancaster G.I., Langley K.G., Berglund N.A., Kammoun H.L., Reibe S., Estevez E., Weir J., Mellett N.A., Pernes G., Conway J.R.W. (2018). Evidence that TLR4 Is Not a Receptor for Saturated Fatty Acids but Mediates Lipid-Induced Inflammation by Reprogramming Macrophage Metabolism. Cell Metab..

[12] Korbecki J., Bajdak-Rusinek K. (2019). The effect of palmitic acid on inflammatory response in macrophages: An overview of molecular mechanisms. Inflamm. Res..

[13] Zhou H.P., Urso C.J., Jadeja V. (2020). Saturated Fatty Acids in Obesity-Associated Inflammation. J. Inflamm. Res..

[14] Charles-Messance H., Mitchelson K.A.J., Castro E.D., Sheedy F.J., Roche H.M. (2020). Regulating metabolic inflammation by nutritional modulation. J. Allergy Clin. Immunol..

[15] Jablonski K.A., Amici S.A., Webb L.M., Ruiz-Rosado J.D., Popovich P.G., Partida-Sanchez S., Guerau-de-Arellano M. (2015). Novel Markers to Delineate Murine M1 and M2 Macrophages. PLoS ONE.

[16] Na Y.R., Jung D., Gu G.J., Seok S.H. (2016). GM-CSF Grown Bone Marrow Derived Cells Are Composed of Phenotypically Different Dendritic Cells and Macrophages. Mol. Cells.

[17] Erlich Z., Shlomovitz I., Edry-Botzer L., Cohen H., Frank D., Wang H.Q., Lew A.M., Lawlor K.E., Zhan Y.F., Vince J.E. (2019). Macrophages, rather than DCs, are responsible for inflammasome activity in the GM-CSF BMDC model. Nat. Immunol..

[18] Mills E.L., O’Neill L.A. (2016). Reprogramming mitochondrial metabolism in macrophages as an anti-inflammatory signal. Eur. J. Immunol..

[19] Zhuang H.D., Lv Q., Zhong C., Cui Y.R., He L.L., Zhang C., Yu J. (2021). Tiliroside Ameliorates Ulcerative Colitis by Restoring the M1/M2 Macrophage Balance via the HIF-1 alpha/glycolysis Pathway. Front. Immunol..

[20] Odegaard J.I., Ricardo-Gonzalez R.R., Goforth M.H., Morel C.R., Subramanian V., Mukundan L., Eagle A.R., Vats D., Brombacher F., Ferrante A.W. (2007). Macrophage-specific PPAR gamma controls alternative activation and improves insulin resistance. Nature.

[21] Kelly B., O’Neill L.A.J. (2015). Metabolic reprogramming in macrophages and dendritic cells in innate immunity. Cell Res..

[22] Vergadi E., Ieronymaki E., Lyroni K., Vaporidi K., Tsatsanis C. (2017). Akt Signaling Pathway in Macrophage Activation and M1/M2 Polarization. J. Immunol..

[23] Jin L.Y., Deng Z.H., Zhang J.Y., Yang C., Liu J.J., Han W.D., Ye P., Si Y.L., Chen G.H. (2019). Mesenchymal stem cells promote type 2 macrophage polarization to ameliorate the myocardial injury caused by diabetic cardiomyopathy. J. Transl. Med..

[24] Vasandan A.B., Jahnavi S., Shashank C., Prasad P., Kumar A., Prasanna J. (2016). Human Mesenchymal stem cells program macrophage plasticity by altering their metabolic status via a PGE(2)-dependent mechanism. Sci. Rep..

[25] Lu D., Xu Y., Liu Q.L., Zhang Q. (2021). Mesenchymal Stem Cell-Macrophage Crosstalk and Maintenance of Inflammatory Microenvironment Homeostasis. Front. Cell Dev. Biol..

[26] Anderson P., Souza-Moreira L., Morell M., Caro M., O’Valle F., Gonzalez-Rey E., Delgado M. (2013). Adipose-derived mesenchymal stromal cells induce immunomodulatory macrophages which protect from experimental colitis and sepsis. Gut.

[27] Souza-Moreira L., Soares V.C., Dias S.D.G., Bozza P.T. (2019). Adipose-derived Mesenchymal Stromal Cells Modulate Lipid Metabolism and Lipid Droplet Biogenesis via AKT/mTOR -PPAR gamma Signalling in Macrophages. Sci. Rep..

[28] Aleshin S., Grabeklis S., Hanck T., Sergeeva M., Reiser G. (2009). Peroxisome Proliferator-Activated Receptor (PPAR)-gamma Positively Controls and PPAR alpha Negatively Controls Cyclooxygenase-2 Expression in Rat Brain Astrocytes through a Convergence on PPAR beta/delta via Mutual Control of PPAR Expression Levels. Mol. Pharmacol..

[29] Lima J.B., Araujo-Santos T., Lazaro-Souza M., Carneiro A.B., Ibraim I.C., Jesus-Santos F.H., Luz N.F., Pontes S.D., Entringer P.F., Descoteaux A. (2017). *Leishmania infantum* lipophosphoglycan induced-Prostaglandin E-2 production in association with PPAR-gamma expression via activation of Toll like receptors-1 and 2. Sci. Rep..

[30] Markovic T., Jakopin Z., Dolenc M.S., Mlinaric-Rascan I. (2017). Structural features of subtype-selective EP receptor modulators. Drug Discov. Today.

[31] Aoki T., Frosen J., Fukuda M., Bando K., Shioi G., Tsuji K., Ollikainen E., Nozaki K., Laakkonen J., Narumiya S. (2017). Prostaglandin E-2-EP2-NF-kappa B signaling in macrophages as a potential therapeutic target for intracranial aneurysms. Sci. Signal..

[32] Wu J.M.F., Cheng Y.Y., Tang T.W.H., Shih C., Chen J.H., Hsieh P.C.H. (2018). Prostaglandin E-2 Receptor 2 Modulates Macrophage Activity for Cardiac Repair. J. Am. Heart Assoc..

[33] Minhas P.S., Latif-Hernandez A., McReynolds M.R., Durairaj A.S., Wang Q., Rubin A., Joshi A.U., He J.Q., Gauba E., Liu L. (2021). Restoring metabolism of myeloid cells reverses cognitive decline in ageing. Nature.

[34] Johansson J.U., Woodling N.S., Wang Q., Panchal M., Hang X.B., Trueba-Saiz A., Brown H.D., Mhatre S.D., Loui T., Andreasson K.I. (2015). Prostaglandin signaling suppresses beneficial microglial function in Alzheimer’s disease models. J. Clin. Investig..

[35] El-Ftesi S., Chang E.I., Longaker M.T., Gurtner G.C. (2009). Aging and Diabetes Impair the Neovascular Potential of Adipose-Derived Stromal Cells. Plast. Reconstr. Surg..

[36] Ciprandi G., Murdaca G., Colombo B.M., De Amici M., Marseglia G.L. (2008). Serum vascular endothelial growth factor in allergic rhinitis and systemic lupus erythematosus. Hum. Immunol..

[37] Mahdy R.A., Nada W.M., Hadhoud K.M., El-Tarhony S.A. (2010). The role of vascular endothelial growth factor in the progression of diabetic vascular complications. Eye.

[38] Han Y.D., Ren J., Bai Y., Pei X.T., Han Y. (2019). Exosomes from hypoxia-treated human adipose-derived mesenchymal stem cells enhance angiogenesis through VEGF/VEGF-R. Int. J. Biochem. Cell Biol..

[39] Becker M., Levings M.K., Daniel C. (2017). Adipose-tissue regulatory T cells: Critical players in adipose-immune crosstalk. Eur. J. Immunol..

[40] Cipolletta D., Feuerer M., Li A., Kamei N., Lee J., Shoelson S.E., Benoist C., Mathis D. (2012). PPAR-gamma is a major driver of the accumulation and phenotype of adipose tissue T-reg cells. Nature.

[41] Cipolletta D., Cohen P., Spiegelman B.M., Benoist C., Mathis D. (2015). Appearance and disappearance of the mRNA signature characteristic of T-reg cells in visceral adipose tissue: Age, diet, and PPAR gamma effects. Proc. Natl. Acad. Sci. USA.

[42] Feuerer M., Herrero L., Cipolletta D., Naaz A., Wong J., Nayer A., Lee J., Goldfine A.B., Benoist C., Shoelson S. (2009). Lean, but not obese, fat is enriched for a unique population of regulatory T cells that affect metabolic parameters. Nat. Med..

[43] Travers R.L., Motta A.C., Betts J.A., Bouloumie A., Thompson D. (2015). The impact of adiposity on adipose tissue-resident lymphocyte activation in humans. Int. J. Obes..

[44] Wouters K., Gaens K., Bijnen M., Verboven K., Jocken J., Wetzels S., Wijnands E., Hansen D., van Greevenbroek M., Duijvestijn A. (2017). Circulating classical monocytes are associated with CD11c(+) macrophages in human visceral adipose tissue. Sci. Rep..

[45] Ivanova-Todorova E., Bochev I., Dimitrov R., Belemezova K., Mourdjeva M., Kyurkchiev S., Kinov P., Altankova I., Kyurkchiev D. (2012). Conditioned Medium from Adipose Tissue-Derived Mesenchymal Stem Cells Induces CD4+FOXP3+Cells and Increases IL-10 Secretion. J. Biomed. Biotechnol..

[46] Song K.J., Cai H.H., Zhang D.M., Huang R.C., Sun D.H., He Y.L. (2018). Effects of human adipose-derived mesenchymal stem cells combined with estrogen on regulatory T cells in patients with premature ovarian insufficiency. Int. Immunopharmacol..

[47] O’Rourke R.W., Gaston G.D., Meyer K.A., White A.E., Marks D.L. (2013). Adipose tissue NK cells manifest an activated phenotype in human obesity. Metab. Clin. Exp..

[48] Boulenouar S., Michelet X., Duquette D., Alvarez D., Hogan A.E., Dold C., O’Connor D., Stutte S., Tavakkoli A., Winters D. (2017). Adipose Type One Innate Lymphoid Cells Regulate Macrophage Homeostasis through Targeted Cytotoxicity. Immunity.

[49] Pierce J.R., Maples J.M., Hickner R.C. (2015). IL-15 concentrations in skeletal muscle and subcutaneous adipose tissue in lean and obese humans: Local effects of IL-15 on adipose tissue lipolysis. Am. J. Physiol. Endocrinol. Metab..

[50] Al-Attar A., Presnell S.R., Clasey J.L., Long D.E., Walton R.G., Sexton M., Starr M.E., Kern P.A., Peterson C.A., Lutz C.T. (2018). Human Body Composition and Immunity: Visceral Adipose Tissue Produces IL-15 and Muscle Strength Inversely Correlates with NK Cell Function in Elderly Humans. Front. Immunol..

[51] Costello R.T., Rey J., Fauriat C., Gastaut J.A., Olive D. (2003). New approaches in the immunotherapy of haematological malignancies. Eur. J. Haematol..

[52] Costa P., Rusconi S., Mavilio D., Fogli M., Murdaca G., Pende D., Mingari M.C., Galli M., Moretta L., De Maria A. (2001). Differential disappearance of inhibitory natural killer cell receptors during HAART and possible impairment of HIV-1-specific CD8 cytotoxic T lymphocytes. Aids.

[53] Wensveen F.M., Jelencic V., Valentic S., Sestan M., Wensveen T.T., Theurich S., Glasner A., Mendrila D., Stimac D., Wunderlich F.T. (2015). NK cells link obesity-induced adipose stress to inflammation and insulin resistance. Nat. Immunol..

[54] Lee B.C., Kim M.S., Pae M., Yamamoto Y., Eberle D., Shimada T., Kamei N., Park H.S., Sasorith S., Woo J.R. (2016). Adipose Natural Killer Cells Regulate Adipose Tissue Macrophages to Promote Insulin Resistance in Obesity. Cell Metab..

[55] Li Y., Wang F.J., Imani S., Tao L., Deng Y.C., Cai Y. (2021). Natural Killer Cells: Friend or Foe in Metabolic Diseases?. Front. Immunol..

[56] Lee H., Da Silva I.P., Palendira U., Scolyer R.A., Long G.A.V., Wilmott J.S. (2021). Targeting NK Cells to Enhance Melanoma Response to Immunotherapies. Cancers.

[57] La Cava A. (2017). Leptin in inflammation and autoimmunity. Cytokine.

[58] Song J.F., Deng T. (2020). The Adipocyte and Adaptive Immunity. Front. Immunol..

[59] Achari A.E., Jain S.K. (2017). Adiponectin, a Therapeutic Target for Obesity, Diabetes, and Endothelial Dysfunction. Int. J. Mol. Sci..

[60] Tong H.V., Luu N.K., Son H.A., Hoan N.V., Hung T.T., Velavan T.P., Toan N.L. (2017). Adiponectin and pro-inflammatory cytokines are modulated in Vietnamese patients with type 2 diabetes mellitus. J. Diabetes Investig..

[61] Francisco V., Pino J., Campos-Cabaleiro V., Ruiz-Fernandez C., Mera A., Gonzalez-Gay M.A., Gomez R., Gualillo O. (2018). Obesity, Fat Mass and Immune System: Role for Leptin. Front. Physiol..

[62] Wrann C.D., Laue T., Hubner L., Kuhlmann S., Jacobs R., Goudeva L., Nave H. (2012). Short-term and long-term leptin exposure differentially affect human natural killer cell immune functions. Am. J. Physiol. Endocrinol. Metab..

[63] Jahn J., Spielau M., Brandsch C., Stangl G.I., Delank K.S., Bahr I., Berreis T., Wrann C.D., Kielstein H. (2015). Decreased NK cell functions in obesity can be reactivated by fat mass reduction. Obesity.

[64] Artis D., Spits H. (2015). The biology of innate lymphoid cells. Nature.

[65] O’Sullivan T.E., Rapp M.Y., Fan X.Y., Weizman O.E., Bhardwaj P., Adams N.M., Walzer T., Dannenberg A.J., Sun J.C. (2016). Adipose-Resident Group 1 Innate Lymphoid Cells Promote Obesity-Associated Insulin Resistance. Immunity.

[66] Wang H.D., Shen L., Sun X.T., Liu F.C., Feng W.H., Jiang C.P., Chu X.H., Ye X., Jiang C., Wang Y. (2019). Adipose group 1 innate lymphoid cells promote adipose tissue fibrosis and diabetes in obesity. Nat. Commun..

[67] Molofsky A.B., Nussbaum J.C., Liang H.E., Van Dyken S.J., Cheng L.E., Mohapatra A., Chawla A., Locksley R.M. (2013). Innate lymphoid type 2 cells sustain visceral adipose tissue eosinophils and alternatively activated macrophages. J. Exp. Med..

[68] Brestoff J.R., Kim B.S., Saenz S.A., Stine R.R., Monticelli L.A., Sonnenberg G.F., Thome J.J., Farber D.L., Lutfy K., Seale P. (2015). Group 2 innate lymphoid cells promote beiging of white adipose tissue and limit obesity. Nature.

[69] Lee M.W., Odegaard J.I., Mukundan L., Qiu Y.F., Molofsky A.B., Nussbaum J.C., Yun K.R., Locksley R.M., Chawla A. (2015). Activated Type 2 Innate Lymphoid Cells Regulate Beige Fat Biogenesis. Cell.

[70] Everaere L., Yahia S.A., Boute M., Audousset C., Chenivesse C., Tsicopoulos A. (2018). Innate lymphoid cells at the interface between obesity and asthma. Immunology.

[71] Virtanen K.A., Lidell M.E., Orava J., Heglind M., Westergren R., Niemi T., Taittonen M., Laine J., Savisto N.J., Enerback S. (2009). Brief Report: Functional Brown Adipose Tissue in Healthy Adults. N. Engl. J. Med..

[72] Rodriguez A., Ezquerro S., Mendez-Gimenez L., Becerril S., Fruhbeck G. (2015). Revisiting the adipocyte: A model for integration of cytokine signaling in the regulation of energy metabolism. Am. J. Physiol. Endocrinol. Metab..

[73] Qiu Y.F., Nguyen K.D., Odegaard J.I., Cui X.J., Tian X.Y., Locksley R.M., Palmiter R.D., Chawla A. (2014). Eosinophils and Type 2 Cytokine Signaling in Macrophages Orchestrate Development of Functional Beige Fat. Cell.

[74] Fischer K., Ruiz H.H., Jhun K., Finan B., Oberlin D.J., van der Heide V., Kalinovich A.V., Petrovic N., Wolf Y., Clemmensen C. (2017). Alternatively activated macrophages do not synthesize catecholamines or contribute to adipose tissue adaptive thermogenesis. Nat. Med..

[75] Rajbhandari P., Thomas B.J., Feng A.C., Hong C., Wang J.X., Vergnes L., Sallam T., Wang B., Sandhu J., Seldin M.M. (2018). IL-10 Signaling Remodels Adipose Chromatin Architecture to Limit Thermogenesis and Energy Expenditure. Cell.

[76] Villarroya F., Cereijo R., Gavalda-Navarro A., Villarroya J., Giralt M. (2018). Inflammation of brown/beige adipose tissues in obesity and metabolic disease. J. Int. Med..

[77] Schulz T.J., Huang P., Huang T.L., Xue R.D., McDougall L.E., Townsend K.L., Cypess A.M., Mishina Y., Gussoni E., Tseng Y.H. (2013). Brown-fat paucity due to impaired BMP signalling induces compensatory browning of white fat. Nature.

[78] Ravussin Y., Xiao C.Y., Gavrilova O., Reitman M.L. (2014). Effect of Intermittent Cold Exposure on Brown Fat Activation, Obesity, and Energy Homeostasis in Mice. PLoS ONE.

[79] Keipert S., Lutter D., Schroeder B.O., Brandt D., Stahlman M., Schwarzmayr T., Graf E., Fuchs H., de Angelis M.H., Tschop M.H. (2020). Endogenous FGF21-signaling controls paradoxical obesity resistance of UCP1-deficient mice. Nat. Commun..

[80] Ziegler A.K., Damgaard A., Mackey A.L., Schjerling P., Magnusson P., Olesen A.T., Kjaer M., Scheele C. (2019). An anti-inflammatory phenotype in visceral adipose tissue of old lean mice, augmented by exercise. Sci. Rep..

[81] Whitehead A., Krause F.N., Moran A., MacCannell A.D.V., Scragg J.L., McNally B.D., Boateng E., Murfitt S.A., Virtue S., Wright J. (2021). Brown and beige adipose tissue regulate systemic metabolism through a metabolite interorgan signaling axis. Nat. Commun..

[82] Scheele C., Nielsen S. (2017). Metabolic regulation and the anti-obesity perspectives of human brown fat. Redox Biol..

[83] Bettini S., Favaretto F., Compagnin C., Belligoli A., Sanna M., Fabris R., Serra R., Dal Pra C., Prevedello L., Foletto M. (2019). Resting Energy Expenditure, Insulin Resistance and UCP1 Expression in Human Subcutaneous and Visceral Adipose Tissue of Patients with Obesity. Front. Endocrinol..

[84] Lim J., Park H.S., Kim J., Jang Y.J., Kim J.H., Lee Y., Heo Y. (2020). Depot-specific UCP1 expression in human white adipose tissue and its association with obesity-related markers. Int. J. Obes..

[85] Wu D., Molofsky A.B., Liang H.E., Ricardo-Gonzalez R.R., Jouihan H.A., Bando J.K., Chawla A., Locksley R.M. (2011). Eosinophils Sustain Adipose Alternatively Activated Macrophages Associated with Glucose Homeostasis. Science.

[86] Wueest S., Rapold R.A., Schumann D.M., Rytka J.M., Schildknecht A., Nov O., Chervonsky A.V., Rudich A., Schoenle E.J., Donath M.Y. (2010). Deletion of Fas in adipocytes relieves adipose tissue inflammation and hepatic manifestations of obesity in mice. J. Clin. Investig..

[87] Choi E.W., Lee M., Song J.W., Kim K., Lee J., Yang J., Lee S.H., Kim I.Y., Choi J.H., Seong J.K. (2020). Fas mutation reduces obesity by increasing IL-4 and IL-10 expression and promoting white adipose tissue browning. Sci. Rep..

[88] Matsui Y., Tomaru U., Miyoshi A., Ito T., Fukaya S., Miyoshi H., Atsumi T., Ishizu A. (2014). Overexpression of TNF-alpha converting enzyme promotes adipose tissue inflammation and fibrosis induced by high fat diet. Exp. Mol. Pathol..

[89] Kern L., Mittenbuhler M.J., Vesting A.J., Ostermann A.L., Wunderlich C.M., Wunderlich F.T. (2019). Obesity-Induced TNF alpha and IL-6 Signaling: The Missing Link between Obesity and Inflammation-Driven Liver and Colorectal Cancers. Cancers.

[90] Chen Y.Y., Tian Z.G. (2020). Roles of Hepatic Innate and Innate-Like Lymphocytes in Nonalcoholic Steatohepatitis. Front. Immunol..

[91] Martinez-Chantar M.L., Delgado T.C., Beraza N. (2021). Revisiting the Role of Natural Killer Cells in Non-Alcoholic Fatty Liver Disease. Front. Immunol..

[92] Michelet X., Dyck L., Hogan A., Loftus R.M., Duquette D., Wei K., Beyaz S., Tavakkoli A., Foley C., Donnelly R. (2018). Metabolic reprogramming of natural killer cells in obesity limits antitumor responses. Nat. Immunol..

[93] Cuff A.O., Sillito F., Dertschnig S., Hall A., Luong T.V., Chakraverty R., Male V. (2019). The Obese Liver Environment Mediates Conversion of NK Cells to a Less Cytotoxic ILC1-Like Phenotype. Front. Immunol..

[94] Luci C., Vieira E., Perchet T., Gual P., Golub R. (2019). Natural Killer Cells and Type 1 Innate Lymphoid Cells Are New Actors in Non-alcoholic Fatty Liver Disease. Front. Immunol..

[95] Tarantino G., Citro V., Balsano C., Capone D. (2021). Age and Interleukin-15 Levels Are Independently Associated with Intima-Media Thickness in Obesity-Related NAFLD Patients. Front. Med..

[96] Racanelli V., Rehermann B. (2006). The liver as an immunological organ. Hepatology.

[97] Yokota S., Yoshida O., Dou L., Spadaro A.V., Isse K., Ross M.A., Stolz D.B., Kimura S., Du Q., Demetris A.J. (2015). IRF-1 Promotes Liver Transplant Ischemia/Reperfusion Injury via Hepatocyte IL-15/IL-15R alpha Production. J. Immunol..

[98] Cepero-Donates Y., Rakotoarivelo V., Mayhue M., Ma A., Chen Y.G., Ramanathan S. (2016). Homeostasis of IL-15 dependent lymphocyte subsets in the liver. Cytokine.

[99] Zahran W.E., El-Dien K.A.S., Kamel P.G., El-Sawaby A.S. (2013). Efficacy of Tumor Necrosis Factor and Interleukin-10 Analysis in the Follow-up of Nonalcoholic Fatty Liver Disease Progression. Indian J. Clin. Biochem..

[100] Velho S., Paccaud F., Waeber G., Vollenweider P., Marques-Vidal P. (2010). Metabolically healthy obesity: Different prevalences using different criteria. Eur. J. Clin. Nutr..

[101] Smith G.I., Mittendorfer B., Klein S. (2019). Metabolically healthy obesity: Facts and fantasies. J. Clin. Investig..

[102] Pereira S., Teixeira L., Aguilar E., Oliveira M., Savassi-Rocha A., Pelaez J.N., Capettini L., Diniz M.T., Ferreira A., Alvarez-Leite J. (2014). Modulation of adipose tissue inflammation by FOXP3+ Treg cells, IL-10, and TGF-beta in metabolically healthy class III obese individuals. Nutrition.

[103] Proto J.D., Doran A.C., Gusarova G., Yurdagul A., Sozen E., Subramanian M., Islam M.N., Rymond C.C., Du J., Hook J. (2018). Regulatory T Cells Promote Macrophage Efferocytosis during Inflammation Resolution. Immunity.

[104] Sharma M., Schlegel M.P., Afonso M.S., Brown E.J., Rahman K., Weinstock A., Sansbury B.E., Corr E.M., van Solingen C., Koelwyn G.J. (2020). Regulatory T Cells License Macrophage Pro-Resolving Functions During Atherosclerosis Regression. Circ. Res..

[105] Beppu L.Y., Mooli R.G.R., Qu X.Y., Marrero G.J., Finley C.A., Fooks A.N., Mullen Z.P., Frias A.B., Sipula I., Xie B.X. (2021). Tregs facilitate obesity and insulin resistance via a Blimp-1/IL-10 axis. JCI Insight.

[106] Brovkina O., Nikitin A., Khodyrev D., Shestakova E., Sklyanik I., Panevina A., Stafeev I., Menshikov M., Kobelyatskaya A., Yurasov A. (2019). Role of MicroRNAs in the Regulation of Subcutaneous White Adipose Tissue in Individuals with Obesity and without Type 2 Diabetes. Front. Endocrinol..

[107] Zou M.L., Chen Z.H., Teng Y.Y., Liu S.Y., Jia Y., Zhang K.W., Sun Z.L., Wu J.J., Yuan Z.D., Feng Y. (2021). The Smad Dependent TGF-beta and BMP Signaling Pathway in Bone Remodeling and Therapies. Front. Mol. Biosci..

[108] Menke A., Casagrande S., Geiss L., Cowie C.C. (2015). Prevalence of and Trends in Diabetes Among Adults in the United States, 1988–2012. JAMA.

[109] Takeno K., Tamura Y., Kawaguchi M., Kakehi S., Watanabe T., Funayama T., Furukawa Y., Kaga H., Yamamoto R., Kim M. (2016). Relation Between Insulin Sensitivity and Metabolic Abnormalities in Japanese Men with BMI of 23-25 kg/m^2^. J. Clin. Endocrinol. Metab..

[110] Kadowaki S., Tamura Y., Someya Y., Takeno K., Kaga H., Sugimoto D., Kakehi S., Funayama T., Furukawa Y., Suzuki R. (2019). Fatty Liver Has Stronger Association with Insulin Resistance Than Visceral Fat Accumulation in Nonobese Japanese Men. J. Endocr. Soc..

[111] Rodriguez B.L., Curb J.D., Davis J., Shintani T., Perez M.H., Apau-Ludlum N., Johnson C., Harrigan R. (2012). Use of the Dietary Supplement 5-Aminiolevulinic Acid (5-ALA) and Its Relationship with Glucose Levels and Hemoglobin A1C among Individuals with Prediabetes. Clin. Transl. Sci..

[112] Higashikawa F., Noda M., Awaya T., Tanaka T., Sugiyama M. (2013). 5-aminolevulinic acid, a precursor of heme, reduces both fasting and postprandial glucose levels in mildly hyperglycemic subjects. Nutrition.

[113] Saitoh S., Okano S., Nohara H., Nakano H., Shirasawa N., Naito A., Yamamoto M., Kelly V.P., Takahashi K., Tanaka T. (2018). 5-aminolevulinic acid (ALA) deficiency causes impaired glucose tolerance and insulin resistance coincident with an attenuation of mitochondrial function in aged mice. PLoS ONE.

[114] Van Wijk K., Akabane T., Kimura T., Saitoh S., Okano S., Kelly V.P., Takagi M., Kodama K., Takahashi K., Tanaka T. (2021). Heterozygous disruption of ALAS1 in mice causes an accelerated age-dependent reduction in free heme, but not total heme, in skeletal muscle and liver. Arch. Biochem. Biophys..

[115] Okano S., Zhou L.Y., Kusaka T., Shibata K., Shimizu K., Gao X., Kikuchi Y., Togashi Y., Hosoya T., Takahashi S. (2010). Indispensable function for embryogenesis, expression and regulation of the nonspecific form of the 5-aminolevulinate synthase gene in mouse. Genes Cells.

[116] Sugiyama Y., Hiraiwa Y., Hagiya Y., Nakajima M., Tanaka T., Ogura S. (2018). 5-Aminolevulinic acid regulates the immune response in LPS-stimulated RAW 264.7 macrophages. BMC Immunol..

[117] Fujino M., Nishio Y., Ito H., Tanaka T., Li X.K. (2016). 5-Aminolevulinic acid regulates the inflammatory response and alloimmune reaction. Int. Immunopharmacol..

[118] Murakami M., Kamimura D., Hirano T. (2019). Pleiotropy and Specificity: Insights from the Interleukin 6 Family of Cytokines. Immunity.

[119] Ettinger M.P., Littlejohn T.W., Schwartz S.L., Weiss S.R., McIlwain H.H., Heymsfield S.B., Bray G.A., Roberts W.G., Heyman E.R., Stambler N. (2003). Recombinant variant of ciliary neurotrophic factor for weight loss in obese adults—A randomized, dose-ranging study. JAMA.

[120] Zhao J.J., Turpin-Nolan S., Febbraio M.A. (2021). IL-6 family cytokines as potential therapeutic strategies to treat metabolic diseases. Cytokine.

[121] Kraakman M.J., Kammoun H.L., Allen T.L., Deswaerte V., Henstridge D.C., Estevez E., Matthews V.B., Neill B., White D.A., Murphy A.J. (2015). Blocking IL-6 trans-Signaling Prevents High-Fat Diet-Induced Adipose Tissue Macrophage Recruitment but Does Not Improve Insulin Resistance. Cell Metab..

[122] Findeisen M., Allen T.L., Henstridge D.C., Kammoun H., Brandon A.E., Baggio L.L., Watt K.I., Pal M., Cron L., Estevez E. (2019). Treatment of type 2 diabetes with the designer cytokine IC7Fc. Nature.

[123] Lizcano F. (2019). The Beige Adipocyte as a Therapy for Metabolic Diseases. Int. J. Mol. Sci..

[124] Kurylowicz A., Puzianowska-Kuznicka M. (2020). Induction of Adipose Tissue Browning as a Strategy to Combat Obesity. Int. J. Mol. Sci..

